# Mammary Defences and Immunity against Mastitis in Sheep

**DOI:** 10.3390/ani9100726

**Published:** 2019-09-26

**Authors:** Angeliki I. Katsafadou, Antonis P. Politis, Vasia S. Mavrogianni, Mariana S. Barbagianni, Natalia G. C. Vasileiou, George C. Fthenakis, Ilektra A. Fragkou

**Affiliations:** Veterinary Faculty, University of Thessaly, 43100 Karditsa, Greece

**Keywords:** ewe, leucocyte, neutrophils, proteomics, subclinical mastitis, teat, vaccination

## Abstract

**Simple Summary:**

The article reviews the defence mechanisms and the relevant processes that occur in the udder of sheep. Due to the importance of the udder in milk production, animals display many defences to protect the organ. These include the teats, the epithelial and the white-blood cells in the udder, the immunoglobulins and chemical substances that all participate in the various processes. These are influenced by many factors, animal- or management-regulated, which must be taken into account in the formulation of prevention schemes against mastitis in sheep.

**Abstract:**

The objectives of this review paper are to present udder defences, including teat of the udder, mammary epithelial cells, leucocytes, immunoglobulins, complement system and chemical antibacterial agents, to describe cooperation and interactions between them and to elaborate on potentials regarding their significance in mammary immunisation strategies. The teat of the udder provides initial protection to the mammary gland. The mammary epithelial cells synthesise antibacterial proteins and the leucocytes produce various inflammation mediators (cytokines or chemokines), phagocytose bacteria and recognise antigenic structures. In the mammary gland, four immunoglobulins (IgG1, IgG2, IgM and IgA) have important roles against bacterial pathogens. The complement system is a collection of proteins, participating in the inflammatory process through various pathways. Other components contributing to humoral mammary defence include lactoferrin, lysozyme and the lactoperoxidase/myeloperoxidase systems, as well as oligosaccharides, gangliosides, reactive oxygen species, acute phase proteins (e.g., haptoglobin and serum amyloid A), ribonucleases and a wide range of antimicrobial peptides. Management practices, genetic variations and nutrition can influence mammary defences and should be taken into account in the formulation of prevention strategies against ovine mastitis.

## 1. Introduction

Many specific or non-specific mechanisms of varying significance and involvement, which include humoral and cellular immune processes, are responsible for the defence of the mammary gland. Non-specific or specific defence mechanisms active in the mammary gland are part of the innate or adaptive immune systems, respectively. The innate immune system would function to provide the initial defence against invading pathogens, preceding adaptive immunity. The adaptive immune system would respond more efficiently in cases of repeated infections, but less so against new pathogens. Roles and objectives of the two systems are distinct, but, as they share common pathways and mechanisms, they are dependent on each other [1].

The objectives of this review paper were to present mammary defences (teat of the udder, mammary epithelial cells, leucocytes, immunoglobulins and complement system, chemical antibacterial agents) and to describe cooperation and interactions between them.

## 2. The Teat of the Udder

Initial protection of the mammary gland against infections is provided by the teat of the udder. Healthy teats efficiently protect ewes against mastitis by providing the first line of defence against bacteria invading into the mammary gland during lamb suckling, as well as during milking of ewes [2,3]. Fatty acids present on the teat skin have bacteriostatic properties and can thus limit bacterial numbers around the teat orifice and on the teat surface [4]. The keratinised and covered with hydrophobic lipids internal lining of the teat canal entraps invading bacteria, which are then flushed out at the following milking along with teat canal epithelium during the first outflow of milk (“keratin flush”) [1,4]. Teat closure after milking, effected by the local teat musculature, is paramount for inhibiting bacterial entrance. However, some closure is achieved 20 to 30 minutes after completion of milking; during that period animals should be prevented from lying down, as this predisposes to increased exposure of their teats to bacteria [5,6]. It is of note that total closure is not achieved until two hours post-milking [7,8,9]. It is hence recommended that, after milking, animals are walked to clean areas in the farm, with feed available in troughs; this practice would contribute to reducing mammary infections. Specifically, during the dry-period, accumulation of keratin at the teat orifice seals the teat preventing bacterial entrance.

**Figure 1:**
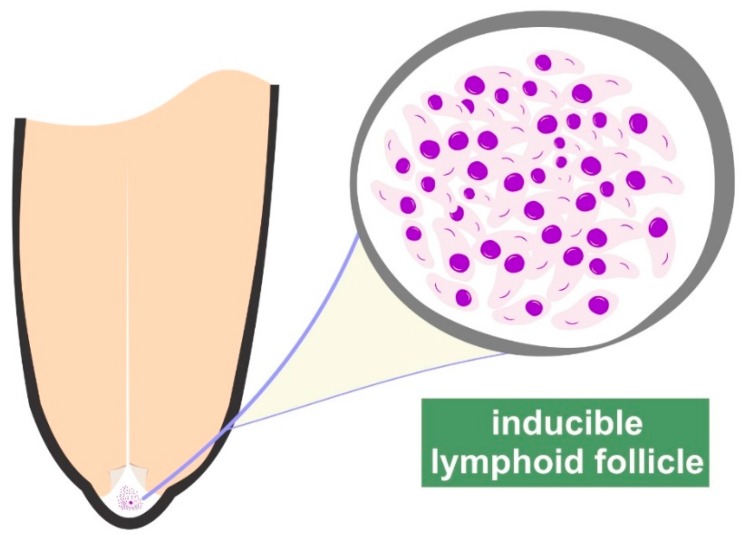
Graphic illustration of the inducible lymphoid follicles in the teat of ewes.

Further, the inducible lymphoid nodules, present at the border between teat duct and teat cistern, play a pivotal role in udder defence [3,10] (Figure 1, Supplementary Materials: Figure S1). These nodules comprise B and T lymphocytes and major histocompatibility complex (MHC) MHC-II^+^ cells, constituting a local organised lymphocytic response. Moreover, presence of *γδ* T lymphocytes therein also indicates their role in bridging the innate and acquired immune responses in the ovine udder [11]. Mucosal-associated lymphoid tissue is an accumulation of effector lymphoid cells previously primed in lymphoid organs and is particularly prominent following repeated infections. The presence of B cells within the follicular structures of the teat indicates an immunological response within the teat end; in addition, the macrophages and the cells expressing MHC-class II cells promote local antigen recognition, presentation and cellular activation. Fragkou et al. [12] have identified active lymphoid follicles even in the teats of pre-pubertal ewes (i.e., animals that had not lactated and thus had not been exposed to intramammary infections) and discussed the possibility of that occurring consequently to an antigenic stimulation in other follicles of similar nature in the body of those animals, e.g., in the bronchioles.

By using culture-independent methods for microbial identification (proteomics methodologies, specifically MALDI-TOF), the existence of microbiota within the teat and the lactiferous ducts of the udder of cows has been reported; a possible source of these bacteria can be the intestine, as it has been found that bacteria from the gut microbiota reach the mammary gland by leucocytes (e.g., dendritic cells, macrophages, lymphocytes) [13]. In an extensive field study, Vasileiou et al. [14] have reported that prevalence of mammary carriage in ewes was 10.0%. The significance of mammary carriage (defined as the presence of bacteria in the udder with no increased somatic cell numbers, i.e., in the absence of inflammation [15,16]) is that the bacteria can play a protective role against invading pathogens, as well as potentially becoming pathogenic under the effect of various factors, which decrease defensive efficacy of hosts or promote pathogenicity of bacteria.

In ewes, Fragkou et al. [17] have concluded that coagulase-negative staphylococci present in high numbers within the teat duct could afford some protection against invading pathogens. Bacterial populations interact among themselves and constitute a ’community‘, where each species contributes to its stability. Many mechanisms by which bacteria and their interactions prevent the invasion and colonisation of pathogenic microorganisms, have been proposed, but are not all fully understood. Occupation of the host’s epithelial surfaces by members of the microbiota and, thus, prevention of pathogen adherence on these cells can be particularly important [18], given that adherence on mammary epithelial cells by the various pathogens is necessary for their multiplication and thereafter for biofilm formation (e.g., staphylococci [19]) or toxin production (e.g., *Mannheimia haemolytica* [20]). Bacterial competition is the situation, during which two bacterial populations compete for multiplication and survival, often resulting in greater bacterial number reduction or lower growth rate than if the two populations were separate [21]. Production of antagonistic substances by bacterial flora and competition for necessary nutritional substances between microbiota and invading organisms [22] can also contribute. Moreover, staphylococci recovered from bovine milk samples have been reported to secrete bacteriocins, leading, under in vitro conditions, to reducing growth of other pathogens [23]. The potential protective role of mammary microbiota can be explained on bacterial interactions. Nevertheless, it may also be proposed that these organisms elicit defence mechanisms, which thus contribute to a more efficient defensive process exhibited by the teat. For example, inflammation mediators may be present in the teat as the result of bacterial carriage infection; these may facilitate the local cellular response and contribute to efficient host defence [12]. Indeed, Rainard and Poutrel [24] have reported that new infections were less frequent in mammary glands already harbouring bacteria therein; in ewes, this has been corroborated by Fragkou et al. [17].

After bacteria have by-passed the teat duct, a cascade of events forms the immunological response of the mammary gland and determines the outcome of the elicited inflammatory responses [25].

## 3. Cells in the Mammary Gland

### 3.1. Mammary Epithelial Cells

The possible role of the epithelia in the initiation of a defence response and the regulation of its initial steps has been studied mainly during the last 20 years [26]. In the case of mammary epithelial cells, this role includes many facets, which can contribute in the effective elimination of invading intramammary pathogens.

As part of a sensory and recognition function of the mammary epithelial cells, Petzl et al. [27] reported in cases of mastitis, in cows, the presence of increased Toll-Like Receptors 2 (TLR2) on their apical side, in contrast to the small amounts found in healthy glands.

Moreover, epithelial cells may also perform some degree of phagocytosis, which is performed by means of formation of membrane ruffles, which enfold the invading bacteria, fuse and form a pathogen containing vesicle [26]. Thereafter, the phagosome matures by fusion and fission of endocytic vesicles [28], which is further accompanied by acidification of the lumen mediated by early recruitment of vacuolar ATPases and formation of the phagolysosome. Subsequent phagocytosis mechanisms are similar to those occurring in leucocytes [26]. In ewes, the changes occurring in the cytoskeleton of mammary epithelial cells as the result of bacterial invasion, have been depicted in the significant number of proteins related to cell reorganisation and biogenesis identified in ovine milk samples collected immediately after intramammary infection [29,30,31]. Nevertheless, differences have been identified in the response of the mammary epithelial cells against various pathogens: in cows, a strong response has been identified against *Escherichia coli*, but a weak and delayed one against *Staphylococcus aureus* and *Streptococcus uberis* [32,33].

A further role (possibly the main one) has been described by the results of studies performed in cows [34,35] or goats [36], which have shown that the mammary epithelial cells responded to intramammary infections by generating a variety of inflammatory mediators: cytokines (e.g., interleukin-8), chemokines (e.g., CCL5 [Chemokine (C-C motif) ligand]) and *β*-defensins. Also in cows, Eckersall [37,38] and Smolenski [39] have reported the production of haptoglobin and serum amyloid from the mammary epithelial cells. Evidence of production of antimicrobial proteins by mammary epithelial cells has also been reported by McDonald et al. [40], based on the identification of an isoform of serum amyloid in the colostrum of ewes, which they termed “mammary-associated serum amyloid”. Katsafadou et al. [31], who worked in ewes by using a proteomics approach, have further suggested the production by mammary epithelial cells of proteins involved in the inflammation process, namely serum amyloid, haptoglobin, but not of alpha-1 antiproteinase, as reported in does [36]. Cathelicidin is synthesised in mammary epithelial cells and immediately released upon exposure of these cells to the invading pathogens [41]. Indeed, Cubeddu et al. [41] reported that cathelicidin production occurs before leucocyte influx into the mammary gland.

An additional role of the host’s innate immunity during mammary infections has been suggested during the study of ’pathogen-associated molecular patterns‘ (PAMPs), which are the result of repeated exposure to microbial pathogens or their constituents. Endotoxin tolerance is such a mechanism, which is defined as the changes in responsiveness by the host to bacterial endotoxin challenge, following an initial encounter with endotoxin [42,43]. The process has been mainly investigated in humans and mice, especially in connection with septic shock. There can be a scope for studying it also in ruminants, given the importance of Gram-negative bacteria (in ewes, *M. haemolytica* mainly), as well as that ruminants are more sensitive to endotoxin than laboratory animals [44]. Although the underlying molecular mechanisms of the process have not been fully characterised, PAMPs-mediated endotoxin tolerance is characterised by a temporary inactivation of the induction of inflammatory genes by TLRs [45]. Endotoxin tolerance should be considered as a mechanism potentially protecting against septic shock, through the inhibition of systemically harmful factors [46]. Monocytes and dendritic cells are the cell types involved in endotoxin tolerance [42,47]. The significance of the system as part of mechanisms against mastitis has been investigated and relevant experimental findings have lent support to that hypothesis [45,48,49]. The theory implies that Gram-negative mastitis pathogens contain several highly potent PAMPs, which can trigger multiple pathogen recognition receptors (PRRs) in the infected animal during an intramammary infection. For example, *E. coli* antigens can be identified more easily by mammary PRRs or virulence factors of Gram-negative pathogens have a higher potency to trigger a strong local immune response. During and after contact with PAMPs (e.g., lipopolysaccharide), a temporary ’reprogramming‘ seems to occur in the mammary tissue, which affects its response [50]. Günther et al. [51] have tested the effectiveness of a variety of candidate substances for priming mammary epithelial cells to respond and have reported that induction of cross-tolerance through lipopeptides or other TLR2 ligands might be possible, thus confirming that bacterial antigenic substances are able to induce the beneficial features of endotoxin tolerance in mammary epithelial cells. In ewes, in which *M. haemolytica* rather than *E. coli*, is a significant pathogen [52], no relevant studies have been published; however, the issue is discussed given that Hughes and Watson [53] have reviewed the comparative aspects of the mammary microenvironment in various mammalian species as part of a one-health approach. In contrast, in the case of *S. aureus*, which is an important mammary pathogen in ewes [52], activation of the mammary gland in its entirety was found to be necessary for clearance of the infection [49].

### 3.2. Leucocytes

In healthy mammary glands, all types of leucocytes are present [54,55,56,57,58] (Table 1). Leucocytes originate from the blood circulation and are, thus, referred to as ‘somatic cells’. 

The number of leucocytes in healthy mammary glands of ewes has not been firmly established. Many authors have proposed various thresholds for leucocytes in the milk of healthy ewes. Moreover, factors other than infection (e.g., stage of lactation) can also influence leucocyte numbers in normal milk and these should be taken into account [62]. Possibly, the best approach is the one suggested by Berthelot et al. [56], who proposed a dynamic approach based on the use of two thresholds able to discriminate healthy (cell counts < 0.5 × 10^6^ cells mL^-1^ on each of two samples) from infected (somatic cell counts > 1.0 × 10^6^ cells mL^-1^ on each of two samples) ewes, coupled with bacteriological examinations in samples found with cell numbers between those two thresholds. There is also a conflict regarding the proportion of epithelial cells in the milk of healthy ewes, with findings ranging widely. Paape et al. [63] indicated that the proportion of epithelial cells was 1% to 2% of all cells in milk, whilst Boutinaud and Jammes [60] reported that epithelial cells in the milk of ewes were at “very low levels”. In sharp contrast to the above findings, Leitner et al. [64] have indicated that epithelial cells constituted ∼80% of the cells in the milk of healthy ewes.

Macrophages are the first among the leucocytes present in the mammary gland to counteract the invading pathogens and to initiate the defensive leucocytic response [65,66]; they produce various defence-related components, which include cytokines and chemokines (e.g., tumour necrosis factor, interleukin 8; Table 2), as well as antimicrobial proteins and peptides (e.g., *β*-defensins and cathelicidins). These cells contribute to the induction of specific local responses, through antigen processing and presentation to lymphocytes, in association with MHC class II [67]. As a result, within two to four hours after infection, there is influx of blood constituents, e.g., leucocytes, into mammary tissues and, hence, into the milk [10,56,68,69]. Increased permeability of the blood–milk barrier by various mechanisms (e.g., by modulating claudins at the tight mammary junctions) allows blood constituents and molecules to enter the infected mammary gland. Moreover, the diameter of mammary vessels and blood volume therein increase soon after infection, leading to the transportation of an increased amount of blood constituents into the mammary gland [70,71].

Hence, leucocytes (initially neutrophils) enter into the infected mammary gland and proteins also leak into the milk [28,29]. That way, the inflammatory response is developed and sustained. The reverse phenomenon can also occur: molecules and, more importantly, pathogens that had not been eliminated by the mammary defences, may pass from the mammary gland into the blood circulation, with effects of bacteraemia (in case of bacterial entrance from mammary gland into blood) or endotoxaemia (in case of entrance of bacterial toxins) [29,72]. Influx of neutrophils into the mammary gland (Figure S2) results from simultaneous function of various pathways and the participation of several molecules, e.g., selectins and integrins, which regulate chemotactic activity of leucocytes [67,73,74]. Antigens of invading pathogens are processed in macrophages and B lymphocytes and appear on the membranes in association with MHC class I or II; that way, they may be recognised by different lymphocytes [75].

Interferon-*γ* contributes in upregulation of MHC-I expression and MHC-II antigen presentation, increasing the recognition of extraneous (or foreign) peptides by cytotoxic T cells and inducing the activation of T helper cells. As soon as antigens are recognised, activation of T helper cells occurs; then, these produce cytokines participating in the activation and polarisation of B and T lymphocytes and macrophages. The intramammary influx of leucocytes during infection, coupled with the desquamation of mammary epithelial cells, lead to an increase of leucocyte numbers in milk. This is an established criterion for diagnosis of subclinical mastitis [54,55,56,76]. Roles of the various leucocyte subsets are distinct, but interdependent. Further, increase of leucocytes in milk during infection results in clot formation in milk, which can be detected ultrasonographically (Figure S3) and is used for diagnosis of subclinical mastitis [77,78]. Neutrophils phagocytose bacteria and proceed to perform intracellular killing by rapid release of reactive oxygen species: superoxide radicals and hydrogen peroxide (‘respiratory burst’) [79]. Neutrophils also release various proteins with clear antibacterial activity [73]; among these, a vital role is played by cathelicidins [29,73], which are implicated in intracellular bacterial killing [80,81], as they destroy the lipoprotein membrane of microbes; another antibacterial protein released by neutrophils is S100-A9 protein [73]. Measurement of cathelicidin in milk has also been found to improve diagnosis of subclinical mastitis in ewes [29]. Further, macrophages are also involved in the defence process and participate in bacterial killing, as well as securing a continuing immune response [81]. Lymphocytes recognise a variety of antigenic structures via membrane receptors, which define their specificity, diversity and memory characteristics [82]. According to their molecular type, two different T cell subsets have been characterised: *αβ* and *γδ* T cells; in milk, *αβ* T lymphocytes are present in higher numbers than *γδ* T lymphocytes—these latter cells, are more numerous in milk than in blood [83]. It has also been reported that lymphocytes subsets in milk were different than in blood; T cytotoxic lymphocytes prevail over T helper lymphocytes in milk, which determine an inversed CD4^+^/CD8^+^ (CD: cluster of differentiation) ratio compared to blood [83]. During intramammary infections (Figure S4), preferential trafficking of T or B lymphocytes and natural killer cells regulates specific and non-specific immunological responses.

Before infection, in the mammary gland *αβ* T cells prevail, predominantly exhibiting the CD8^+^ phenotype, which attributes cytotoxic or suppressor functions [84]. CD3^+^, present on all T lymphocytes, is responsible for transducing intracellularly the message of T-cell receptor and antigen-MHC complex binding. CD4^+^ (T helper) cells produce a variety of immunoregulatory cytokines following antigen recognition with MHC-II molecules. As mentioned above, in sheep *γδ* T lymphocytes are more abundant in mammary parenchyma and lacteal secretions compared to blood [83]; these cells can mediate cytotoxicity, with variable involvement of MHC molecules and, in addition, play a role in antibacterial immunity, particularly in mucosae. 

Efficiency of phagocytic killing can determine the severity of the disease to be developed, i.e., from subclinical to haemorrhagic clinical mastitis [85]. Early expression of the various inflammatory reaction modulators also plays a role in the final severity of the disease, as it has been associated with reduced numbers of pathogens in the mammary parenchyma [85].

**Figure 2:**
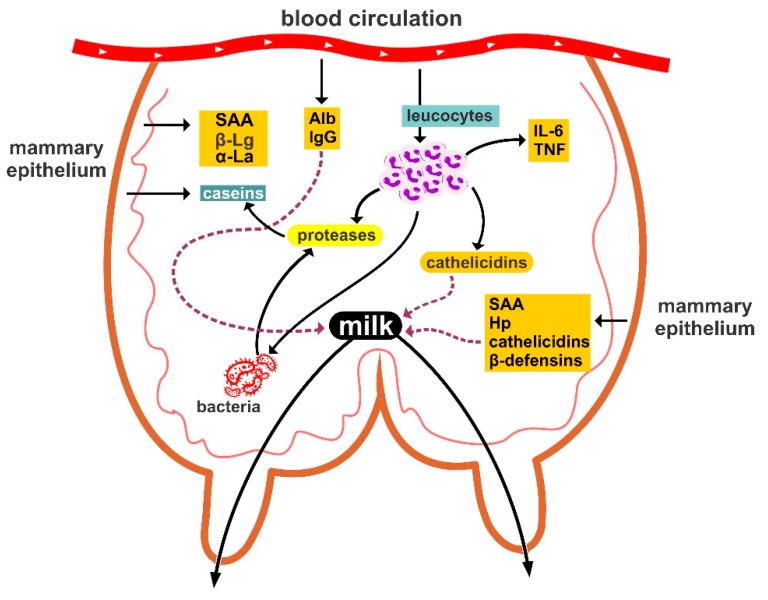
Graphic illustration of the main defensive mechanisms in the mammary gland of ewes. Alb: albumins, Hp: haptoglobin, IL-6: interleukin-6, SAA: serum amyloid A, TNF: tumour necrosis factor, *α*-La: *α*-lactalbumin, *β*-Lg: *β*-lactoglobulin.

A graphic illustration of the main defensive mechanisms in the mammary gland is in Figure 2.

## 4. Immunoglobulins and Complement System

### 4.1. Immunoglobulins

Immunoglobulins are the most important specific soluble humoral factors, present in colostrum and milk. Four different classes of immunoglobulins have important roles in the mammary gland against bacterial pathogens: IgG1, IgG2, IgM and IgA. Immunoglobulins may leak into the mammary gland from blood (IgG1), may be produced locally by antigen-activated plasma cells (IgA, IgM) or may appear by either pathway (IgG2) [86,87,88]. Wellnitz et al. [89] have demonstrated that increase of IgG1 and IgG2 in the mammary gland is a controlled and compound-specific process, following various patterns and not exclusively an unspecific type of leakage. Low level selective transport of plasma derived IgE into mammary secretion of the ovine mammary gland, which may be augmented by low level local production of IgE in the gland, has been also demonstrated [90].

In general, IgG1, IgG2 and IgM act in opsonising bacteria, by means of which the invading pathogens are identified and ’presented‘ to the leucocytes (neutrophils, macrophages) for phagocytosis and, hence, efficient destruction [91]. Further, these immunoglobulins exhibit many functions, including complement fixation, prevention of adhesion of pathogenic microbes to endothelial lining, inhibition of bacterial metabolism by blocking enzymes, agglutination of bacteria and neutralisation of toxins and viruses [92]. IgM antibodies, which are produced in smaller amounts than IgG, are considerably more efficient than IgG in most activities, especially in complement fixation [93]. Sheep colostrum has been found to exhibit higher IgM concentration compared to that of cattle or goats [94]. The significance of opsonisation is easily understood, as without it phagocytosis would be unsystematic and erratic, hence leading to inefficient defence. Nevertheless, it is noted that in milk bacteria are not opsonised by antibodies for subsequent phagocytosis as efficiently as in blood. Finally, IgA acts in bacterial agglutination, which contributes in limiting bacterial dissemination and colonisation.

### 4.2. Complement System

Complement is a collection of proteins produced mainly by the liver, as well as by macrophages and monocytes; for component 3 (C3), a local synthesis in the mammary gland has also been suggested [95]. The complement effector molecules, circulating in serum and interstitial fluids, exist largely in precursor states that are activated rapidly in a proteolytic and cascade-like fashion, following recognition of pathogen-associated molecular patterns and/or noxious self-derived danger-associated molecular patterns [96]. Complement can be activated systemically in the blood, via three main routes: (i) the classical pathway, through which uncoated or immunoglobulin-coated antigens are recognised, (ii) the lectin pathway, triggered by the recognition of microbial carbohydrates through mannose binding lectin, collectins or ficolins followed by activation of the mannose-binding lectin-associated serine proteases and (iii) the alternative complement pathway (also termed the amplification pathway, as it perpetuates complement activation initiated by the classical and/or lectin pathways), characterised by tonic low-level C3 hydrolysis to C3(H_2_O) [96].

In the mammary gland, the system participates in the immune defence, being involved in evoking and controlling the inflammatory process, in participating in bacterial opsonisation and presentation, in recruiting leucocytes and even in direct killing of pathogens [97,98]. In healthy mammary glands, the complement system is activated only through the alternative complement pathway; the classical pathway is not functional, as C1q component is absent or present in smaller concentrations than in blood [95]. During inflammation, complement dependent bactericidal and opsonic activities increase in blood [28,99] (Figure S5). Pathogens opsonised by IgG or complement have increased affinity to phagocytose Fc or complement C3b receptors, respectively, promoting phagocyte–bacteria attachment and bacterial phagocytosis. Yet, in the mammary gland, the alternative complement pathway is not as efficient as in other tissues. In cows, neutrophils within the mammary gland have been found to be less efficient than in blood in the phagocytosis of even opsonised bacteria [95].

Attachment of bacteria to phagocytes is necessary for phagocytosis and, subsequently, intracellular microbiocidal function. Nevertheless, some pathogens, including staphylococci, are able to, sometimes, survive within the leucocytes. These bacteria may be killed by phagocytes activated with lymphokines; these are produced by sensitised lymphocytes challenged with specific antigen and induce rapid attraction of leucocytes to lesions. *S. aureus* strain-specific pathogenicity underscores the importance of pathogen factors in progression of infectious process [100].

## 5. Chemical Antibacterial Agents

Other components contributing to humoral mammary defence include lactoferrin, lysozyme and the lactoperoxidase/myeloperoxidase systems. These participate in immunity modulation, by playing a role in bacteria opsonisation and neutrophil phagocytic activity and performing transcriptional activation of various molecules. Other biochemical components therein, which are further parts of the inflammatory response, include oligosaccharides, gangliosides, reactive oxygen species, acute phase proteins (e.g., haptoglobin and serum amyloid A), ribonucleases and a wide range of antimicrobial peptides.

### 5.1. Lactoferrin

Lactoferrin is an iron-binding glycoprotein, mainly produced by the mammary epithelial cells and in smaller quantities by neutrophils [101,102]. Its expression in milk (Figure S6) is inversely related to alveolar development. The antib acterial effect of lactoferrin is expressed mainly in the epithelium lining of the ducts and cisterns, but not at the proximal end of the teat canal [103]. It is greatly enhanced in increased bicarbonate ion concentrations and reduced concentrations of the lactoferrin inhibitor, citrate ions, which are present during the dry period. Lactoferrin exerts a bacteriostatic effect mainly by competing with bacteria for available iron or by binding to bacterial surfaces [104]; hence, it is of particular significance against Gram-negative bacteria (e.g., *M. haemolytica*, a primary cause of ovine mastitis [52]). Its function includes alteration of integrity and permeability of bacterial cell wall and sensitisation of bacteria to antimicrobial agents. Also, it contributes to bacterial killing and promotes adhesion and aggregation of neutrophils to the endothelial surface, as well as being involved in activation of the complement system via the alternative pathway and in antigen-processing by cells of the reticuloendothelial system and in antibody production [4]. Mean lactoferrin concentrations in infected ovine mammary glands have been shown to be up to 4.8 times higher than those in healthy ones, demonstrating its role in the natural defence mechanisms against mammary gland inflammation in sheep [105]. Moreover, recent work performed in ewes, has indicated that presence of lactoferrin genotype AA was associated with lower prevalence of subclinical mastitis [106].

### 5.2. Lysozyme

Lysozyme derives from blood or is synthesised in the mammary gland from leucocytes during intramammary infections and has inhibitory or lytic activity mainly against Gram-positive bacteria [4]. It is noteworthy that lysozyme exerts its antibacterial action in synergy with antibodies, complement and lactoferrin, increasing susceptibility of bacteria to the various defence mechanisms and functions. In ewes, the potential protective role of lysozyme for the mammary gland has been indicated in a study, in which its concentration was found to be higher in uninfected mammary glands than in glands with mastitis [58]. Further, lysozyme has been found to contribute in the regulation of the inflammatory response and in the immune homeostasis on the epithelial surfaces by activating regulatory T lymphocytes [107,108].

### 5.3. Lactoperoxidase

Lactoperoxidase is locally synthesised in the mammary gland, in the presence of thiocyanate of hepatic origin and hydrogen peroxide of bacterial or endogenous origins [109]. It exerts its antibacterial activity through the formation of activated oxygen products, e.g., hypothiocyanate, a metabolite enhancing bactericidal activity of leucocytes. Under in vitro conditions, the system has been found with significant antibacterial properties, which, in clinical situations, show similar efficacy only during the dry-period; its role during the lactation period is limited, likely because of interference by other milk proteins (Figure S6). Possibly also, concentrations of thiocyanate ions in milk would depend on nutritional regime and small oxygen tension in the mammary gland might act as an inhibitor for production of hydrogen peroxide; these two factors may further account for the limited efficacy of the system against mastitis causing pathogens in lactating animals.

## 6. Modulation of Mammary Defences

### 6.1. Factors Affecting Mammary Defences

Various factors that may affect mammary defences are relevant to various facets of mammary defences and immunity and act to enhance or hinder immunity at different levels. Therefore, they are of importance in the design of mastitis prevention schemes, as well as in the investigation of flock mastitis problems.

Chapped teats, caused by physical causes (e.g., low temperatures) or chemical agents (e.g., disinfectants), predispose ewes to mastitis, as the result of bacterial accumulation on teat skin and compromised antibacterial properties [110,111,112,113]. Further, efficient function of the inducible lymphoid nodules at the teat duct can be compromised by physical (e.g., chaps) or microbial (e.g., viral infections) disorders of teats, which thus predispose animals to mastitis [114,115]. Hence, maintenance of teat health should be an integral part of all mastitis prevention programs in ewes. Moreover, factors related to teat morphology can also play a role in development of mammary infections, e.g., short or wide teats can facilitate bacterial entrance into the mammary parenchyma.

Genetic differences in the susceptibility of ewes to mastitis have been reported; indeed, mastitis is considered a disease amenable for genetic studies. Differences in the susceptibility to mastitis have been associated with particular breeds of sheep [116], as well as with somatic cell counts [56,117]. Possibly, polymorphism of MHC genes, which has been detected in sheep [84], may be related to susceptibility of mastitis, although there is only limited evidence about that. In any case, selection for mastitis resistance based on decreased somatic cell counts has been shown to be of value in selection of individuals with reduced susceptibility to mastitis [118,119,120]. A recent study has indicated that genes differentially expressed in mammary tissues of ewes with clinical mastitis, were involved with regulation of the immune response and the inflammation procedure in these animals [121]; this finding directly associates genetic background of ewes with mammary defences.

Nutrition can play a role in regulation of mammary defences and immunity. Energy has been recognised as an important factor in promoting phagocytosis and intracellular killing of bacteria by leucocytes; in this context, it has been found that ewes with pregnancy toxaemia were at increased risk of developing mastitis in the immediately post-partum period [122]. Selenium is possibly the most studied nutrient with regards to immunoregulatory effect [123]. In brief, selenium is a component of glutathione peroxidase, which can play a protective role against reactive oxygen species damaging various cellular components within leucocytes and thus hindering intracellular killing, therefore reduced selenium intake can lead to impaired leucocytic function. Within this frame, the practice of selenium administration in pregnant ewes at the final stage of gestation, which is aimed to prevent selenium deficiency in newborn lambs, may also have an effect in improving post-partum mammary defences of ewes as well; however, there is no direct evidence for that, but only indirect results [123]. To some extent, vitamin E has similar biological properties with selenium and is implicated in at least the same pathways as selenium. Zinc is a component of teat keratin and skin; zinc deficiencies can adversely affect the integrity of the teat duct and thus facilitate bacterial entrance. Vitamin A deficiency has also been suggested to lead in increased risk of mastitis in ewes, as it may compromise integrity of epithelia and hence lead to impaired mammary defence [123,124]. Therefore, it becomes evident that incorrect nutrition may adversely affect animal defences and hence conclude in increased mastitis risk.

### 6.2. Immunisation

Investigation of immunological responses in the mammary gland of ewes against causal organisms and clarification of the role of the various immunological components supports attempts to protect ewes against mastitis, by enhancing immunity and potentiating responses to treatment with antibiotics. For example, it has been shown that local (mammary) administration of immunogens (e.g., inactivated *S. aureus*) in non-lactating ewes enhanced kinetics of neutrophil influx with no involvement of complement in the immunological response. The basis of vaccination is the enhancement of acquired/specific immunity. Vaccination aims to recognise specific determinants of a pathogen that activate a selective response leading to bacterial elimination [125].

Vaccines licenced against ovine mastitis aim mainly to protect animals against staphylococcal mastitis. Most currently licenced traditionally anti-staphylococcal vaccines are preparations of whole cell cultures of the vaccinal strain. These are of older technology and suboptimal efficacy. With regard to adjuvants used in these vaccines, it has been found that deposition of milk complement components on the bacterial surface does not contribute to the defence of the mammary gland. In general, administration of these vaccines would lead mainly in reducing the severity of clinical signs of affected animals, rather than decreasing the incidence of risk of the disease. More recently, biofilm matrix polysaccharides have been used for development of protective immune response against *S. aureus* mastitis in ewes [126]. This approach employs cell-free surface polysaccharide in various vehicles, bacterial unbound cells or bacterial cells embedded in their biofilm matrix in various adjuvants. Vaccination with whole bacterial cells surrounded by matrix with polysaccharide conferred immunity against *S. aureus* mammary infection and mastitis. Nowadays, there is also evidence that this approach also confers immunity against coagulase-negative staphylococcal species, which are the primary cause of subclinical mastitis [127].

Further, it has been recently reported that intramammary administration of a vaccine already licenced against respiratory infections might also offer a protective effect against *M. haemolytica* mastitis [128]. This approach contributed to protection of ewes after experimental challenge through the induction of a Th17 type response; the protective effect was evident at seven but not at 14 days post-challenge. If appropriately improved, such a vaccine may contribute to protecting ewes against *M. haemolytica*, the second most-important causal agent of the disease [52].

## 7. Conclusions

It becomes evident that the defence mechanisms of the mammary gland are complex and act at various levels. Various defences are in place and are applied through many pathways, which are greatly interdependent. The mechanisms can be enhanced or hindered through health management (including immunisation), which thus becomes of significant importance in mastitis prevention.

## Figures and Tables

**Table 1:** Types of leucocytes present in milk of healthy ewes.

Leucocyte Types	Proportion of Total Leucocytes [59,60,61]
Macrophages	40–85%
Neutrophils	5–35%
Lymphocytes	10–20%

**Table 2:** Cytokines involved in mammary defence response.

Cytokine	Main Role
Interleukin-1	Upregulation of neutrophil migration to the mammary gland, increase of neutrophil numbers, promotion of phagocytic activity in the mammary gland.
Interleukin-2	Upregulation of macrophage proliferation in the mammary gland, improvement of lymphocyte antibacterial properties.
Interleukin-8	Upregulation of neutrophil migration to the mammary gland.
Granulocyte colony-stimulating factor	Increase of neutrophil numbers, promotion of phagocytic activity in the mammary gland.
Granulocyte macrophage colony-stimulating factor	Improvement of neutrophil chemotaxis and bactericidal properties, increase of neutrophil numbers in the mammary gland.
Macrophage colony-stimulating factor	Upregulation of macrophage proliferation.
Interferon-*γ*	Promotion of neutrophil phagocytic activity.
Tumour necrosis factor	Enhancement of inflammatory process, promotion of neutrophil phagocytic activity.

## Supplementary Materials

The following are available online at https://www.mdpi.com/2076-2615/9/10/726/s1, Figure S1: (a) Inducible lymphoid nodule, present at the border between teat duct and teat cistern, with presence of lymphocytes (Haematoxylin and eosin [H and E] stain) (Mavrogianni, personal collection); (b) inducible lymphoid nodule, present at the border between teat duct and teat cistern, with presence of T lymphocytes (CD3^+^ [cluster of differentiation]) (immunohistochemical stain) (Fragkou, personal collection); (c) inducible lymphoid nodule, present at the border between teat duct and teat cistern (H and E stain) (Fragkou, personal collection); (d) inducible lymphoid nodule, present at the border between teat duct and teat cistern (immunohistochemical stain) (Fragkou, personal collection). Figure S2: (a) Presence of neutrophils in milk during acute stage of mammary 23 infection (Giemsa stain) (Mavrogianni, personal collection); (b) presence of neutrophils in mammary tissue during acute stage of mammary infection (H and E stain) (Fthenakis, personal collection). Figure S3: Presence of clots within the teat cistern of ewes during mastitis, as a consequence of cell accumulation therein, detected ultrasonographically (longitudinal section, image taken and processed on a MyLab^®^ 30 ultrasonography system (ESAOTE SpA, Italy]) with linear transducer, imaging frequency: 12.0 MHz, scanning depth: 30 mm) (Barbagianni, personal collection). Figure S4: (a) Presence of lymphocytes in mammary tissue during chronic stage of mammary infection (H and E stain) (Fthenakis, personal collection); (b) presence of lymphocytes in teat during chronic stage of mammary infection (immunohistochemical stain) (Fragkou, personal collection). Figure S5: Identification of complement proteins: complement C3 (CO_3_) and complement factor B (CFAB) spots on a two-dimensional agarose gel from blood of a ewe with mastitis (protein identification by MALDI-TOF MS) (Katsafadou, personal collection). Figure S6: Identification of lactoferrin (TRFL) and lactoperoxidase (PERL) spots on a two-dimensional agarose gel from the milk of a ewe with mastitis (protein identification by MALDI-TOF MS) (Katsafadou, personal collection).
