# Supplementary material for: Mammary Defences and Immunity against Mastitis in Sheep
Source: Animals (Basel). 2019 Sep 26;9(10):726. doi: 10.3390/ani9100726 (PMC6826578; doi:10.3390/ani9100726)
Supplement: Supplementary file 1 [file animals-09-00726-s001.pdf]

Review

Mammary defences and immunity against mastitis in sheep

Angeliki I. Katsafadou [1+], Antonis P. Politis [1+], Vasia S. Mavrogianni [1], Mariana S. Barbagianni [1], Natalia G.C. Vasileiou [1], George C. Fthenakis [1*], Ilektra A. Fragkou [1]

+ These authors have contributed equally and their names are listed alphabetically

Figure S1: (a) Inducible lymphoid nodule, present at the border between teat duct and teat cistern, with presence of lymphocytes (H&E stain) (Mavrogianni, personal collection); (b) Inducible lymphoid nodule, present at the border between teat duct and teat cistern, with presence of T lymphocytes (CD3+) (immunohistochemical stain) (Fragkou, personal collection); (c) Inducible lymphoid nodule, present at the border between teat duct and teat cistern (H&E stain) (Fragkou, personal collection); (d) Inducible lymphoid nodule, present at the border between teat duct and teat cistern (immunohistochemical stain) (Fragkou, personal collection).

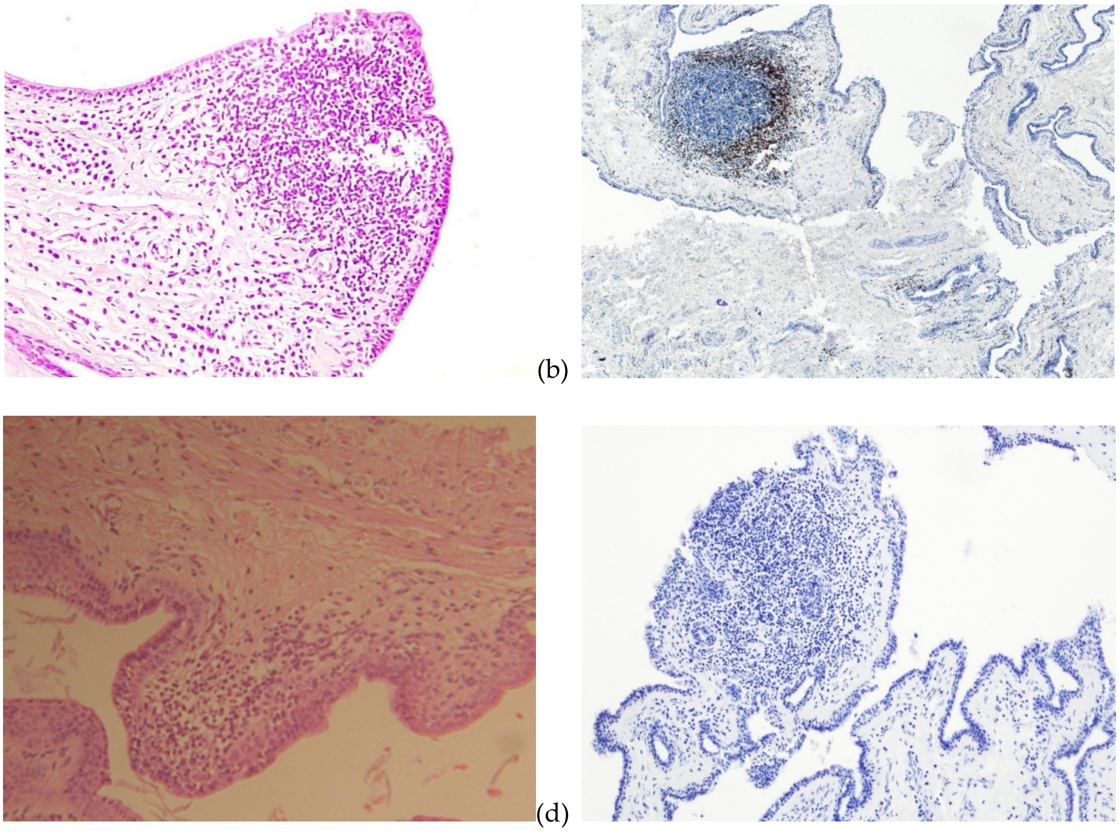

(a) (b) (c) (d)

Figure S2: (a) Presence of neutrophils in milk during acute stage of mammary infection (Giemsa stain) (Mavrogianni, personal collection); (b) Presence of neutrophils in mammary tissue during acute stage of mammary infection (H&E stain) (Fthenakis, personal collection).

(a) 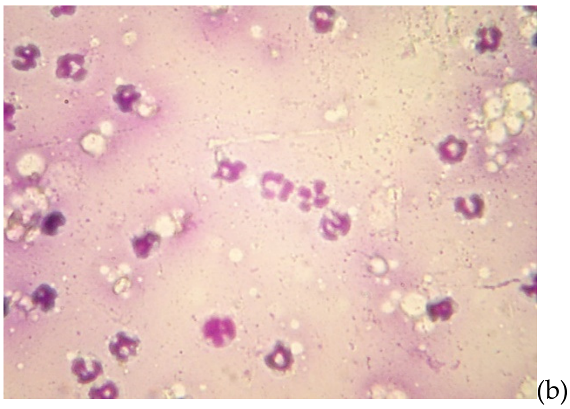 (b) 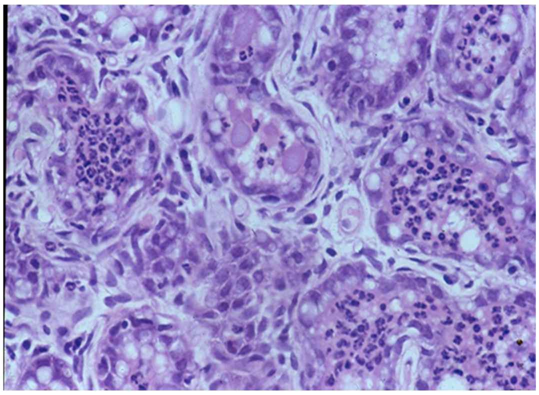

Figure S3: Presence of clots within the teat cistern of ewes during mastitis, as a consequence of cell accumulation therein, detected ultrasonographically (longitudinal section, image taken and processed on a MyLab® 30 ultrasonography system [ESAOTE SpA, Italy] with linear transducer, imaging frequency: 12.0 MHz - scanning depth: 30 mm) (Barbagianni, personal collection).

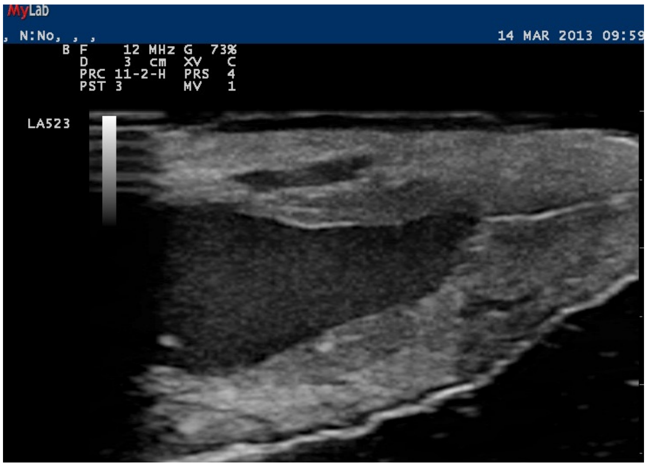

Figure S4: (a) Presence of lymphocytes in mammary tissue during chronic stage of mammary infection (H&E stain) (Fthenakis, personal collection); (b) Presence of lymphocytes in teat during chronic stage of mammary infection (immunohistochemical stain) (Fragkou, personal collection).

(a) 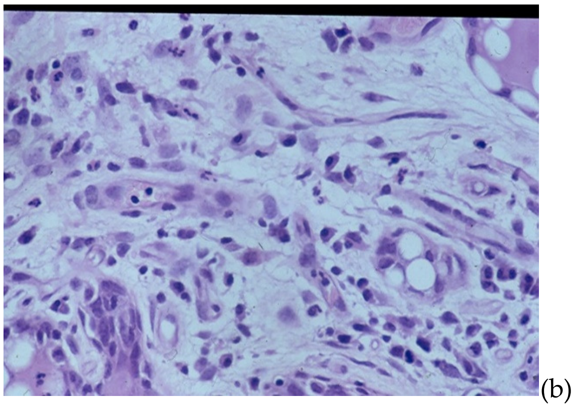 (b) 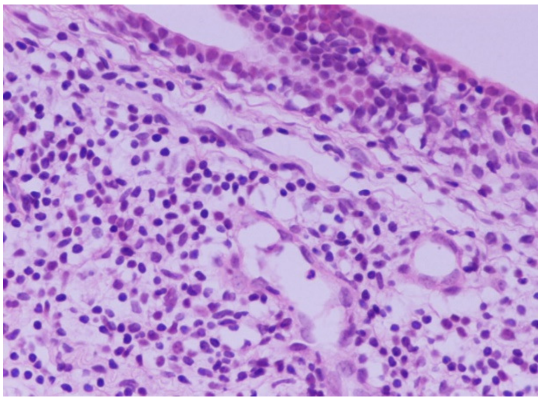

Figure S5: Identification of complement proteins: complement C3 (CO3) and complement factor B (CFAB) spots on a two-dimensional agarose gel from blood of a ewe with mastitis (protein identification by MALDI-TOF MS) (Katsafadou, personal collection).

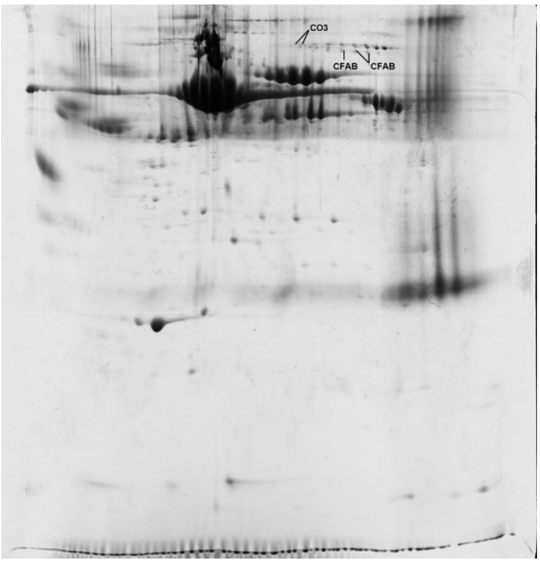

Figure S6: Identification of lactoferrin (TRFL) and lactoperoxidase (PERL) spots on a two-dimensional agarose gel from the milk of a ewe with mastitis (protein identification by MALDI-TOF MS) (Katsafadou, personal collection).

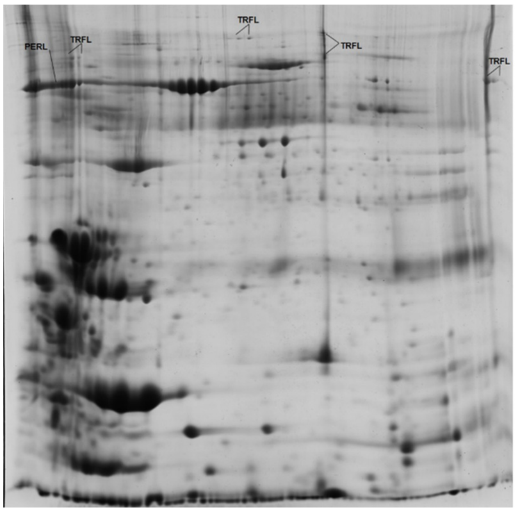

© 2019 by the authors. Submitted for possible open access publication under the terms and conditions of the Creative Commons Attribution (CC BY) license (http://creativecommons.org/licenses/by/4.0/).
